# Sex and age differences in hibernation patterns of common hamsters: adult females hibernate for shorter periods than males

**DOI:** 10.1007/s00360-016-0995-z

**Published:** 2016-05-02

**Authors:** Carina Siutz, Claudia Franceschini, Eva Millesi

**Affiliations:** Department of Behavioral Biology, University of Vienna, Althanstrasse 14, 1090 Vienna, Austria

**Keywords:** Hibernation, Torpor, Sex differences, Timing, Body mass, Common hamster

## Abstract

In this study, we investigated the timing and duration of hibernation as well as body temperature patterns in free-ranging common hamsters (*Cricetus cricetus*) with regard to sex and age differences. Body temperature was recorded using subcutaneously implanted data loggers. The results demonstrate that although immergence and vernal emergence sequences of sex and age groups resembled those of most hibernators, particularly adult females delayed hibernation onset until up to early January. Thus, in contrast to other hibernators, female common hamsters hibernated for shorter periods than males and correspondingly spent less time in torpor. These sex differences were absent in juvenile hamsters. The period between the termination of hibernation and vernal emergence varied among individuals but did not differ between the sex and age groups. This period of preemergence euthermy was related to emergence body mass: individuals that terminated hibernation earlier in spring and had longer euthermic phases prior to emergence started the active season in a better condition. In addition, males with longer periods of preemergence euthermy had larger testes at emergence. In conclusion, females have to rely on sufficient food stores but may adjust the use of torpor in relation to the available external energy reserves, whereas males show a more pronounced energy-saving strategy by hibernating for longer periods. Nonetheless, food caches seem to be important for both males and females as indicated by the euthermic preemergence phase and the fact that some individuals, mainly yearlings, emerged with a higher body mass than shortly before immergence in autumn.

## Introduction

Hibernating animals overcome periods of energy shortage and cold ambient temperatures by expressing heterothermy. This is characterized by multiday torpor bouts with reduced metabolic rate and body temperature, alternating with short arousal intervals at normal body temperature (Buck and Barnes 2000; Geiser 1988b, 2004; Geiser and Ruf 1995; Heldmaier et al. 2004; Heldmaier and Ruf 1992; Ortmann and Heldmaier 2000; Ruf and Geiser 2015; Wang and Hudson 1971). The apparent advantage of hibernation is saving energy (Heldmaier et al. 2004; Heldmaier and Ruf 1992; Levesque and Tattersall 2010; Ruf and Geiser 2015; Ruf and Heldmaier 1992; Wang 1978, 1989), but other benefits, such as reduced water loss and parasite load, and even lower predation risk, have also been documented (Geiser and Brigham 2012). At the same time, this strategy also imposes costs on an individual because torpor is associated with neuronal impairment (Arendt et al. 2003), reduced memory retention (Millesi et al. 2001), immune suppression (Prendergast et al. 2002), oxidative stress (Carey et al. 2000), and cellular damage (Turbill et al. 2013). It has, therefore, been hypothesised that animals adjust the time spent in torpor to available energy reserves (Boyles et al. 2007; French 2000; Humphries et al. 2003a, b; Munro et al. 2005; Vuarin et al. 2015). Accordingly, individuals would minimize the costs of heterothermy by expressing torpor less frequently when energy reserves are sufficient. The hibernation period requires energy storages, which individuals accumulate prior to autumnal immergence either internally as body fat (Florant and Healy 2012; Humphries et al. 2003b; Michener 1992; Sheriff et al. 2013) or externally as food stores (Day and Bartness 2003; French 2000; Humphries et al. 2003b). The latter strategy has been assumed to be preferably used by common hamsters (Eibl-Eibesfeldt 1953; Niethammer 1982), although more recent findings showed pronounced sex differences in foraging behavior (feeding above ground versus food caching) during the active season: males mainly fed above ground, whereas females almost exclusively cached food (Siutz et al. 2012). Correspondingly, adult males had higher body fat proportions shortly before immergence than adult females and probably smaller food caches (Siutz et al. 2012).

Most hibernators, particularly those relying exclusively on body fat, show a strict annual timing of reproduction, prehibernation fattening, and hibernation. Females usually produce one litter per season (Gür and Kart Gür 2005; Kenagy and Barnes 1988; Michener 1998; Millesi et al. 1999a), and immergence into and emergence from the hibernacula often follow typical sex- and/or age-specific patterns (Barnes 1996; Blumstein et al. 2004; Buck and Barnes 1999; Gür and Kart Gür 2005; Kenagy et al. 1989; Michener 1984; Millesi et al. 1999b; Sheriff et al. 2011). The timing of the annual cycle of common hamsters resembles that of other hibernators and follows the general pattern with males emerging from the hibernacula in spring before females (Franceschini and Millesi 2005; Millesi et al. 2004; Ulbrich and Kayser 2004; Weinhold and Kayser 2006). The reproductive period starts shortly after female emergence, but in contrast to most mammalian hibernators, female hamsters can produce up to three litters per season resulting in an extended reproductive period lasting until September in some individuals (Franceschini-Zink and Millesi 2008; Hufnagl et al. 2011; Millesi et al. 2004; Siutz and Millesi 2012). Testes regression in males occurs during August, and shortly thereafter (September), they enter their hibernacula (Lebl and Millesi 2008). Females immerge about 1 month later than males, probably due to their reproductive effort (Franceschini-Zink and Millesi 2008; Hufnagl et al. 2011; Millesi et al. 2004). Juvenile hamsters, finally, start to immerge during October (Franceschini and Millesi 2005).

In some species, such as ground squirrels, eastern chipmunks, or woodchucks, adult males start to hibernate later, show fewer and/or shorter torpor bouts, spend less time in torpor, or end hibernation earlier than adult females (Batavia et al. 2013; Buck et al. 2008; Geiser 1988a; Healy et al. 2012; Kart Gür and Gür 2015; Körtner et al. 2010; Michener 1992; Munro et al. 2005; Sheriff et al. 2011; Young 1990; Zervanos et al. 2013; Zervanos and Salsbury 2003). Such sex differences in hibernation patterns have even been found in juveniles (e.g., 13-lined ground squirrels, Kisser and Goodwin 2012) and were mainly attributed to longer euthermic periods prior to vernal emergence (Michener 1992; Sheriff et al. 2011; Young 1990), autumnal food hoarding, and territory defence (Buck and Barnes 2003; Buck et al. 2008; Healy et al. 2012; Kart Gür and Gür 2015; Michener 1992).

Although the timing of the active season is controlled by an endogenous rhythm entrained by photoperiodic cues, high interspecific variation, e.g., in sciurid rodents suggests that species vary in their ability to alter responses to endogenous and environmental cues. In facultative hibernators, the expression of hibernation is highly variable and can be affected by environmental factors like the availability and quality of food hoards (reviewed in Williams et al. 2014). Previous studies on hibernation in common hamsters under semi-natural conditions (Wassmer 2004; Wollnik and Schmidt 1995) demonstrated a high individual variation in body temperature patterns during winter, ranging from regular, deep torpor bouts alternating with short arousals to irregular patterns with extended euthermic periods, and only a few torpor bouts. Some individuals showed no deep torpor bouts at all (Wassmer 2004; Wollnik and Schmidt 1995) or even exhibited above-ground activity during winter (Wassmer 2004). In these studies, however, food pellets or corn was provided ad libitum outside the hibernaculum and repeatedly supplemented during winter. Under natural conditions, such food resources are usually not available, thus an effect of food supplementation during winter on hibernation patterns and activity outside the burrow cannot be excluded. The aim of this study was to investigate hibernation patterns in free-living common hamsters by comparing the timing and duration of hibernation as well as body temperature patterns and overwinter mass loss between adult males, adult females, and juveniles. In addition, we analyzed relationships between individual hibernation patterns and immergence and emergence body mass.

## Methods

### Field techniques

The study was carried out in urban areas in southern Vienna, Austria, where free-ranging populations of common hamsters inhabit parks and green areas surrounding building complexes. During the active season of common hamsters in Vienna (March/April–October/November), we applied capture-mark-recapture techniques using Tomahawk live traps baited with peanut butter, which were checked at 20-min intervals (Franceschini-Zink and Millesi 2008). Hamsters were released from the traps into cone-shaped cotton sacks laterally equipped with Velcro fasteners, which enabled examining individuals without anaesthesia. For permanent identification, we subcutaneously implanted a transponder (PIT tag, Data Mars) at first capture; for distant recognition, we additionally fur marked each individual in different patterns using commercial hair dye. We documented sex, and age was classified as adult (hibernated at least once) or juvenile (born in the current season; Franceschini-Zink and Millesi 2008; Siutz and Millesi 2012). At each capture, we measured body mass (±1 g) using an electronic scale and determined the individual reproductive status (testes width in males; closed/open vagina and small/swollen teats in females). Conception in females was defined as the date of observed copulations if followed by a body mass increase (indicating gestation) and rapid mass loss after about 3 weeks (indicating parturition). This was paralleled by changes in teat development. Details on this method are described elsewhere (Franceschini et al. 2007). After the investigation (duration 5–10 min), the hamsters were released in front of their burrows, guaranteeing immediate orientation in familiar surroundings. The procedure did not seem to negatively affect the animals, because we found no indications for trap avoidance or changes in behavior, body condition, and survival (Siutz et al. unpublished data). We monitored all hamsters until autumn when they entered their hibernacula, as indicated by trapping and observation failure. To ensure that the animals terminated above-ground activity, we plugged their burrows with leaves and monitored the burrow entrances to detect potential activity. The date when the individual was trapped or observed for the last time in autumn was defined as immergence date, if no activity at the burrow could be detected until spring. Immergence body mass was defined as the individual’s weight measured within 1 week before its immergence into the hibernaculum.

### Hibernation patterns

Body temperature was recorded during three winter seasons (2009/10, 2012/13, 2013/14) using temperature data loggers (iButtons, DS1922L-F5#, range: −40 to +85 °C, accuracy: ±0.5 °C, Maxim Integrated Products International, Dublin, Ireland). The iButtons (coated in Elvax ethylene vinyl acetate resins, DuPont, and paraffin, gas-sterilised; potted mass: ~4.5 g) were implanted subcutaneously in the neck region (dorsal, between the scapulae). This method has proved successful in this species due to the absence of pronounced prehibernation fattening (Hufnagl et al. 2011; Lebl and Millesi 2008). Individuals were trapped in the early morning hours and transported (~20 min) to a veterinary clinic where the implantation was done under isoflurane anaesthesia. When the animals had recovered from anaesthesia, they were returned to the field site and released at their burrows, i.e., about 1–2 h after trapping and still within their daily morning activity period (Schmelzer and Millesi 2005). In total, we implanted 36 hamsters with iButtons. The burrows of the implanted individuals were monitored weekly during winter (open/closed) to detect potential activity. None of the individuals showed signs of above-ground activity until mid-March. Starting in early March, burrows of implanted individuals were checked at daily intervals, active hamsters were recaptured, and the data loggers were removed using the above-mentioned techniques. We were able to recover iButtons of 28 individuals (recovery rate: 78 %). None of the recaptured animals lost the data logger during winter, but two iButtons failed (one of an adult male and one of an adult female). Thus, temperature data of five adult males, seven adult females, eight juvenile males, and six juvenile females were analyzed in this study. Body mass and overwinter survival rates of implanted individuals did not differ from untreated ones of the same age and sex (Siutz et al. unpublished data). Emergence date was defined as the date when an individual was observed above ground or trapped for the first time (this was also the day when an individual removed its burrow plug). Emergence body mass was used for analysis when measured within 1 week after the individual’s vernal emergence date. Mass changes over winter were calculated as percentage of differences between immergence and emergence body mass.

Body temperature was recorded at 90-min intervals from autumn until recapture of individuals in spring. Torpor bouts were defined as multiday periods of reduced body temperature between two arousals, from the sampling interval when body temperature started to continuously decrease from 30 °C to at least 15 °C until it had reached 30 °C again. To analyze hibernation patterns, we defined the following parameters: hibernation onset (date of the first torpor bout), duration of post-immergence euthermy (days spent euthermic after immergence until hibernation onset), number of torpor bouts, mean torpor bout duration (calculated in hours, expressed as days), time spent in torpor (total duration of all torpor bouts, calculated in hours, expressed as days), mean arousal bout duration (calculated in hours, expressed as days), hibernation end (date of the last torpor bout), hibernation duration (days from the onset of the first and termination of the last torpor bout), and duration of preemergence euthermy (days spent euthermic after termination of the last torpor bout until vernal emergence).

### Statistics

Statistical analysis was performed in R (R Development Core Team 2009). We computed linear models for each parameter (Table 1) and included sex, age, and their interaction as predictor variables. To correct for potential annual effects, we initially included the year of data collection as predictor variable in each model, but this parameter had no effect on any hibernation parameter and was also eliminated in all models when minimizing Akaike’s information criterion (AIC). Model residuals were tested for normality using Shapiro–Wilk tests and for homoscedasticity using Levene-tests and were additionally controlled visually by plotting residuals vs. fitted values. ANOVAs from linear models were computed using marginal (Type III) sums of squares. To analyze relationships between hibernation parameters and body mass, we applied Pearson correlations. Significance level was set at *p* ≤ 0.05. Results are shown as mean  ± SE.

**Table 1:** ANOVA (Type III tests) table for effects of sex and age (adult or juvenile) on annual timing and hibernation patterns in common hamsters

Response variable	Predictor variable	Df	*F* value	*p* value
Immergence date	Sex	1	8.405	0.008
	Age	1	0.738	0.399
	Sex × age	1	1.462	0.239
Emergence date	Sex	1	21.563	0.003
	Age	1	2.764	0.405
	Sex × age	1	0.133	0.719
Time inside hibernaculum (d)	Sex	1	0.464	0.505
	Age	1	0.027	0.87
	Sex × age	1	1.113	0.305
Post-immergence euthermy (d)	Sex	1	4.479	0.046
	Age	1	2.619	0.12
	Sex × age	1	2.447	0.132
Hibernation onset (date of first torpor bout)	Sex	1	13.254	0.001
	Age	1	0.449	0.51
	Sex × age	1	4.2	0.052
Hibernation end (date of last torpor bout)	Sex	1	0.081	0.779
	Age	1	3.739	0.066
	Sex × age	1	1.251	0.275
Hibernation duration (d)	Sex	1	9.924	0.005
	Age	1	5.398	0.03
	Sex × age	1	8.596	0.008
Preemergence euthermy (d)	Sex	1	2.048	0.17
	Age	1	2.196	0.156
	Sex × age	1	1.445	0.245
Number of torpor bouts	Sex	1	7.823	0.011
	Age	1	1.334	0.261
	Sex × age	1	6.031	0.022
Time spent in torpor (d)	Sex	1	11.429	0.003
	Age	1	7.586	0.012
	Sex × age	1	10.147	0.004
Torpor bout duration (d)	Sex	1	3.493	0.075
	Age	1	11.134	0.003
	Sex × age	1	4.541	0.045
Arousal bout duration (d)	Sex	1	0.988	0.331
	Age	1	5.978	0.023
	Sex × age	1	3.806	0.064

## Results

### Immergence and emergence sequences and hibernation patterns

**Fig. 1:**
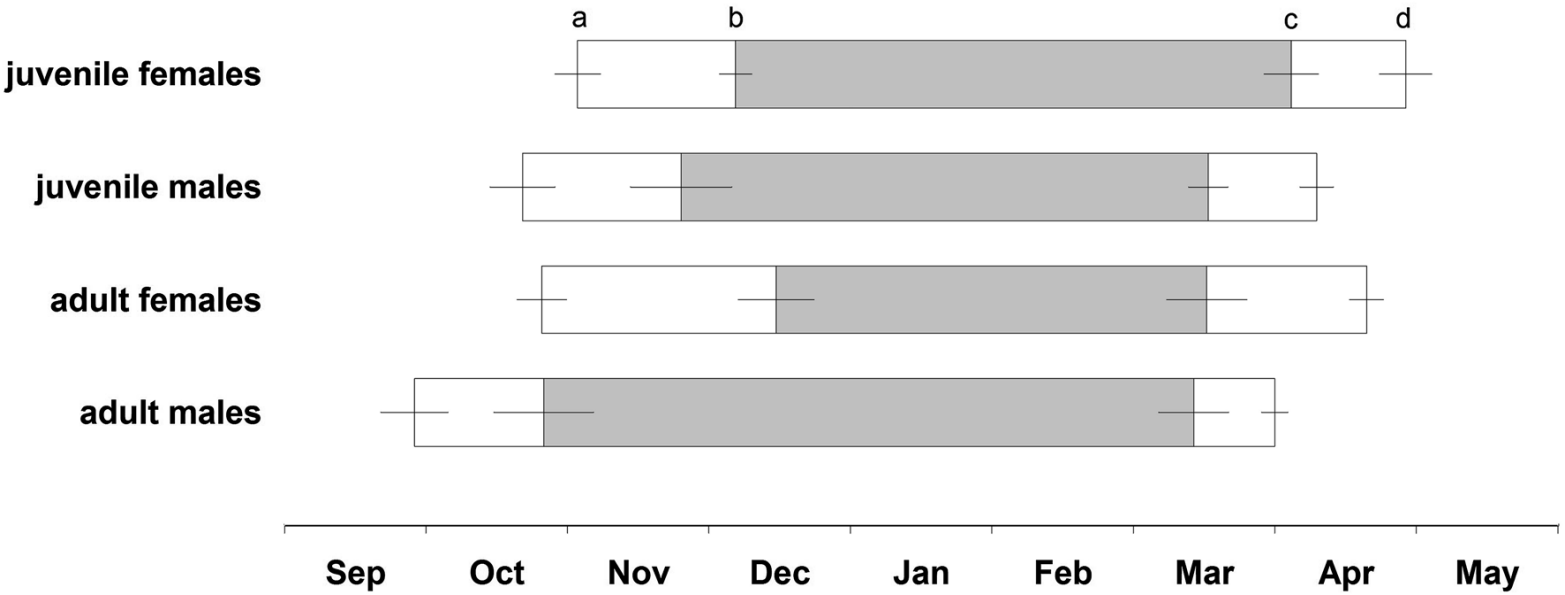
Time spent inside the hibernaculum and duration of hibernation (*gray bars*) in adult males (*n* = 5), adult females (*n* = 7), juvenile males (*n* = 8), and juvenile females (*n* = 6). **a** Autumnal immergence into the hibernaculum (date when an individual terminated above-ground activity), **b** hibernation onset (date of first torpor bout), **c** hibernation end (date of last torpor bout), and **d** vernal emergence from the hibernaculum (date when an individual resumed above-ground activity). Mean ± SE, for statistical results see Table 1

Adult females entered their hibernacula significantly later than adult males, whereas immergence dates were similar in female and male juveniles (Table 1; Fig. 1). In spring, adult females as well as juvenile females (which were now yearlings) left their hibernacula later than males of the respective age group (Table 1; Fig. 1). The time spent inside the hibernaculum was similar among sex and age groups, but shifted in time (Table 1; Fig. 1).

The duration of post-immergence euthermy varied among individuals (Fig. 1, adult males: 11–52 days, adult females: 19–72 days, juvenile males: 10–60 days, juvenile females: 13–43 days) and was significantly longer in adult females than males (Table 1). Correspondingly, adult females started to hibernate later in winter than adult males. They also tended to show their first torpor bout later than juvenile males. Sex differences were absent in juveniles (Table 1; Fig. 1). We found no sex differences among adults in the timing of the last torpor bout, but yearling (former juvenile) females tended to end hibernation later than the other groups (Table 1; Fig. 1). Adult females hibernated for significantly shorter periods than adult males and juveniles (Table 1; Fig. 1). The duration of preemergence euthermy was similar among sex and age groups (Table 1; Fig. 1) and showed a high individual variation (adult males: 10–40 days, adult females: 12–64 days, juvenile males: 11–55 days, juvenile females: 9–42 days).

**Fig. 2:**
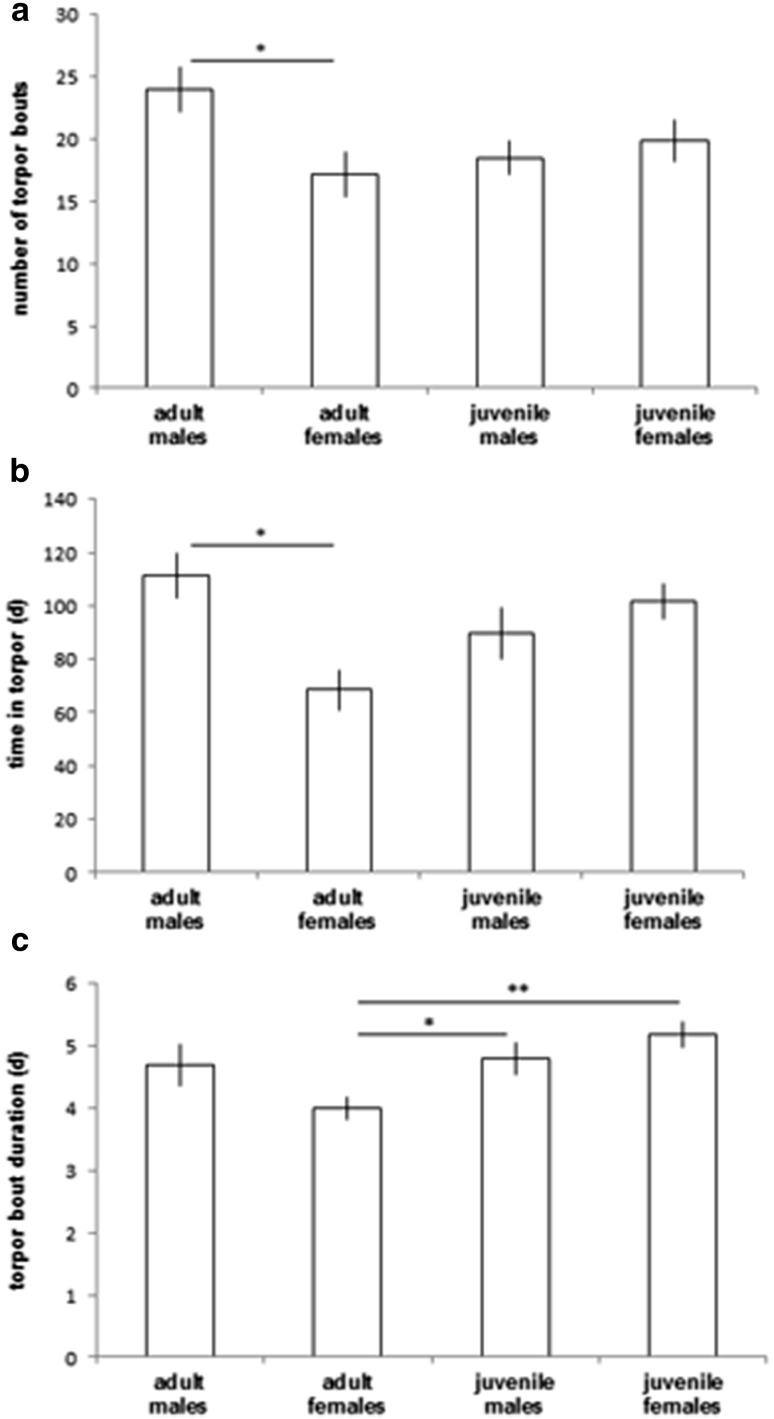
**a** Number of torpor bouts, **b** time spent in torpor, and **c** mean torpor bout duration in adult males (*n* = 5), adult females (*n* = 7), juvenile males (*n* = 8), and juvenile females (*n* = 6). Mean ± SE, *p ≤ 0.05, **p ≤ 0.01, for statistical results see Table 1

Corresponding to the shorter hibernation duration, adult females showed a fewer torpor bouts than adult males (Table 1; Fig. 2a) and also spent less time in torpor compared with the other groups (Table 1; Fig. 2b). Mean torpor bout duration in adult females was significantly shorter than in juveniles, but similar to adult males (Table 1; Fig. 2c). Mean arousal duration did not differ between adult males and females, but juvenile females tended to show the shortest arousal durations (Table 1).

### Conditional effects

**Fig. 3:**
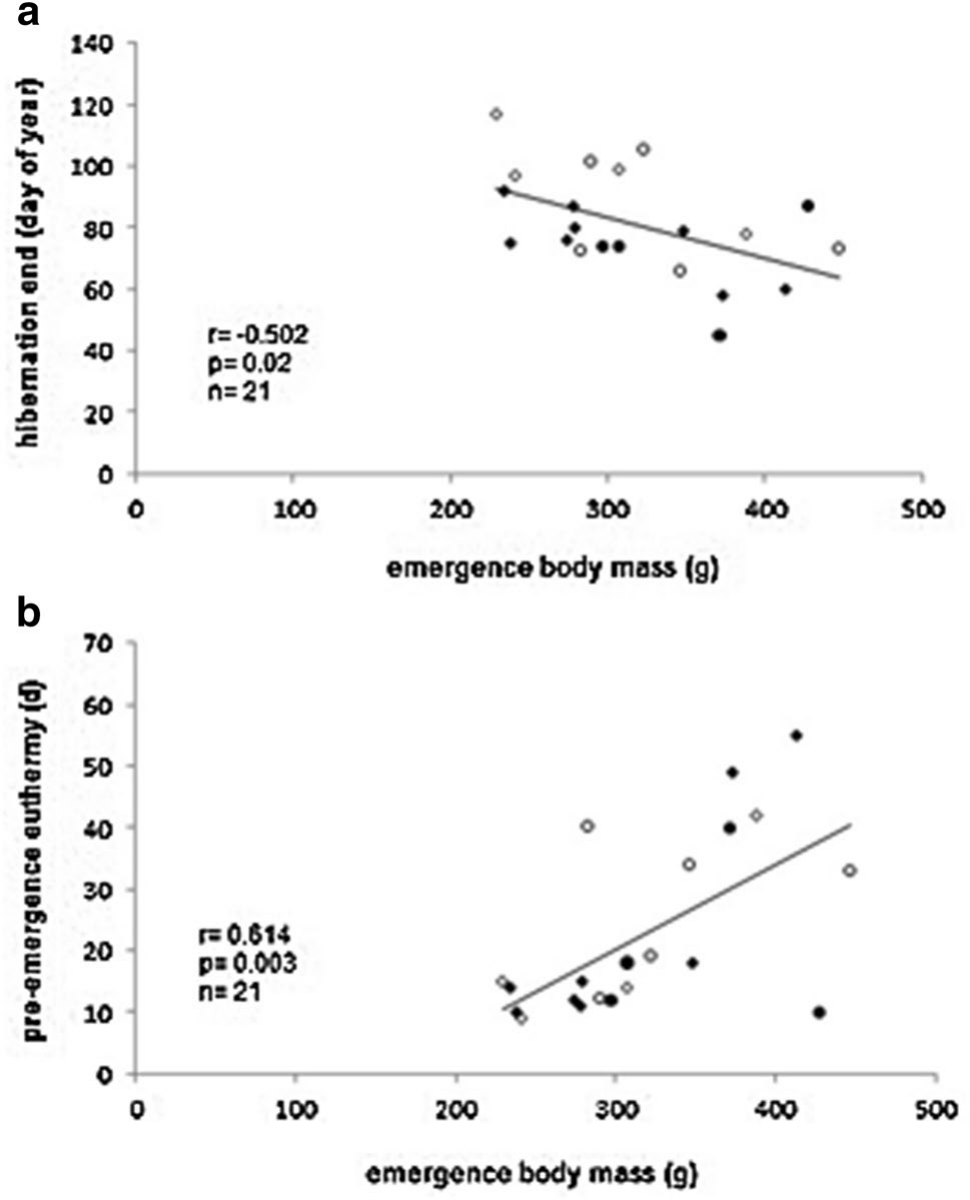
Relationships between emergence body mass and **a** termination of hibernation (day of year 40 refers to Feb 2, day 80 to Mar 21, day 120 to Apr 30), and **b** duration of preemergence euthermy in common hamsters. Age and sex groups are indicated as *black circles* (adult males, *n* = 4), *open circles* (adult females, *n* = 5), *black squares* (juvenile males, *n* = 8), and *open squares* (juvenile females, *n* = 4)

**Fig. 4:**
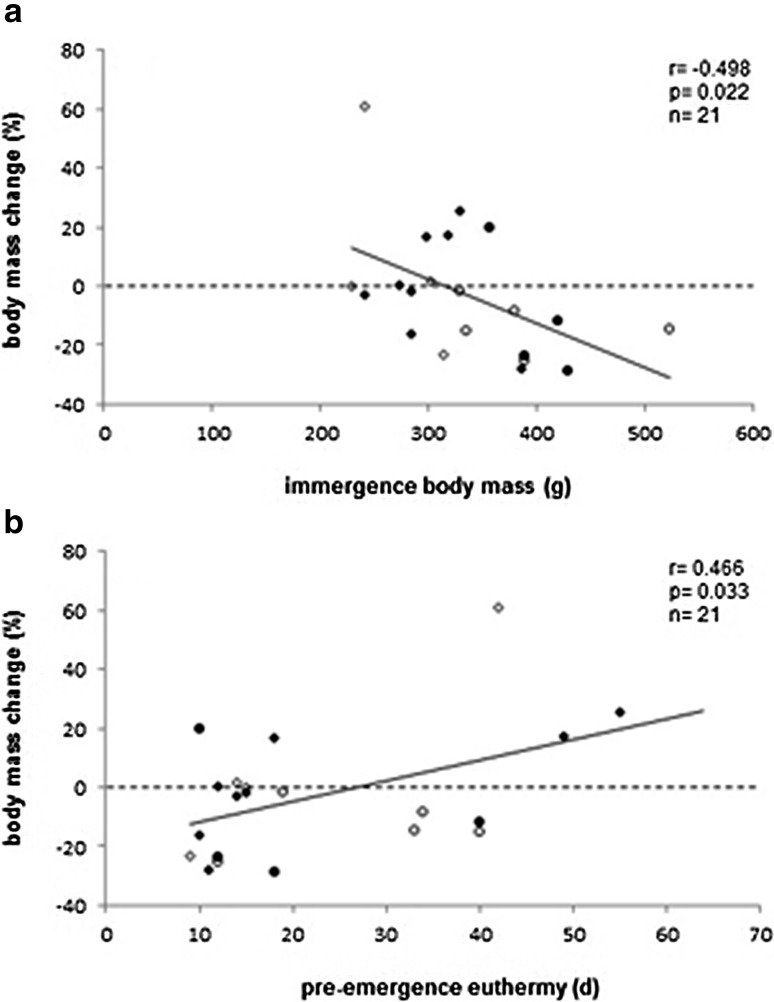
Relationships between body mass changes over winter and **a** immergence body mass, and **b** the duration of preemergence euthermy in common hamsters. Age and sex groups are indicated as *black circles* (adult males, *n* = 4), *open circles* (adult females, *n* = 5), *black squares* (juvenile males, n = 8), and *open squares* (juvenile females, *n* = 4)

Body mass at immergence was related neither to immergence date or the duration of post-immergence euthermy, nor to any of the hibernation parameters. Body mass at vernal emergence was negatively correlated with hibernation end (Fig. 3a) and positively correlated with the duration of preemergence euthermy (Fig. 3b): individuals that terminated hibernation earlier in spring and spent more time in euthermy prior to emergence were heavier at emergence. Body mass changes over winter (%) did not differ between the sexes of either age group (Student’s *t* test, adults: *p* = 0.85, *t* = 0.21 juveniles: *p* = 0.68, *t* = −0.44), but adult individuals tended to lose more mass than juveniles (mass change adults: −12.2 ± 4.9 %, juveniles: 4.2 ± 6.9 %; Student’s *t* test: *p* = 0.067, *t* = −1.93, *n* = 9/12). The percentage of body mass change over winter was negatively related to immergence body mass: hamsters that immerged in autumn with higher body mass lost more weight over winter (Fig. 4a). Furthermore, individuals with longer euthermic periods prior to vernal emergence showed lower mass loss or even gained weight over winter (Fig. 4b) and correspondingly emerged with higher body mass.

In addition, analyses of potential effects of preemergence euthermy on reproduction revealed that males with longer euthermic periods inside their hibernacula had larger testes at emergence (*r* = 0.76, *p* = 0.004, *n* = 12). In females, 92 % (12 of 13) emerged with an open vagina. The timing of conception in spring could be determined in six females. We found no relationship between conception date and the duration of preemergence euthermy (*r* = −0.579, *p* = 0.229, *n* = 6).

## Discussion

All individuals in our study hibernated and showed regular patterns of deep torpor bouts and short arousal episodes for at least a few weeks, and no sign of surface activity during winter was detected. This contrasts with previous reports on common hamsters under semi-natural conditions (Wassmer 2004; Wollnik and Schmidt 1995), probably because those studies provided high-quality food during winter, stimulating the hamsters to leave their hibernacula frequently and collect the food items. The most intriguing results in our study were that adult females hibernated for shorter periods, had a fewer torpor bouts, and spent less time in torpor than adult males. These sex differences were absent in juveniles. This pattern is in contrast to all other documented sex differences in hibernation patterns of mammals, because usually adult males hibernate for shorter periods than females (Batavia et al. 2013; Buck et al. 2008; Geiser 1988a; Healy et al. 2012; Kart Gür and Gür 2015; Körtner et al. 2010; Lee et al. 2016; Michener 1992; Munro et al. 2005; Schmid 1999; Schmid and Kappeler 1998; Sheriff et al. 2011; Young 1990; Zervanos et al. 2013; Zervanos and Salsbury 2003). Divergent patterns, however, were found in bats, which mate before and are pregnant during hibernation (e.g., Grinevitch et al. 1995; Norquay and Willis 2014). Shorter hibernation periods in males are mainly due to earlier termination of heterothermy, longer euthermic periods inside their burrows prior to emergence and earlier vernal emergence than females (e.g., Michener 1992; Munro et al. 2005; Young 1990), presumably to complete spermatogenesis and enhance breeding opportunities (Barnes 1996; Barnes et al. 1986; Gür and Kart Gür 2005; Michener 1983, 1984, 1992; Millesi et al. 1998). Interestingly, vernal emergence sequences of sex and age cohorts in our species followed the common pattern in that males emerged before females, as also shown in previous reports on common hamsters (Franceschini and Millesi 2005; Schmelzer and Millesi 2005; Ulbrich and Kayser 2004; Weinhold and Kayser 2006). We found no sex differences in the timing of hibernation end or in the duration of preemergence euthermy. This indicates that both adult males and females spent up to several weeks euthermic before emergence without leaving the burrow and, thus, had to feed on their remaining food caches.

Delayed immergence into the hibernaculum and thus later hibernation onset were also assumed to contribute to sex differences in hibernation durations. In some ground squirrel species, such as Richardson’s (Michener 1992), golden mantled (Healy et al. 2012), Anatolian (Kart Gür and Gür 2015), and arctic ground squirrels (Buck and Barnes 1999; Buck et al. 2008), males delayed autumnal immergence and entry into torpor to build up food caches (to be consumed in spring during preemergence euthermy) and defend a territory. In our study, autumnal immergence sequences were similar to most hibernating species, with adult males entering their hibernacula before adult females and juveniles. This is because adult females have to wean their offspring, and the juveniles need time for growth before they can start to prepare for hibernation (Michener 1978, 1984; Millesi et al. 2008). The duration of post-immergence euthermy, however, was highly variable among sex and age cohorts and was longer in adult females than in adult males. Later immergence and in particular longer periods of post-immergence euthermy consequently resulted in later hibernation onset in adult females, ranging from November 15 to January 9. Thus, the shorter hibernation period in adult females was due to a delayed onset, while the time spent in the hibernaculum was similar in all sex and age groups. Such a delay could, on the one hand, be shown by light individuals that have to accumulate body fat reserves after immergence (given that food stores are available) before entering the first torpor bout; or alternatively, torpor might be avoided by individuals with sufficient energy reserves. For example, woodchucks (*Marmota monax*) with higher body mass prior to immergence spent less time in torpor during the heterothermic period than lighter ones (Zervanos et al. 2013). Eastern chipmunks (*Tamiasstriatus*) provided with additional food spent less time in torpor than unsupplemented ones (French 2000; Humphries et al. 2003a). In our study, however, body mass at immergence was related neither to the duration of post-immergence euthermy nor to any of the hibernation parameters. The later onset of hibernation in adult females could, therefore, be related to the quantity and/or quality of food stores. Behavioral observations in common hamsters indicated that adult females accumulate more external energy reserves than adult males (Siutz et al. 2012). When foraging above ground, adult males spent about 80 % of the time actually feeding, whereas adult females rarely fed outside their burrows but spent 96 % of the time caching food items and carrying them into their burrows (Siutz et al. 2012). Considering male mating strategies, this sex difference in foraging behavior appeared to be adaptive: adult males changed their burrows frequently until August to monitor receptive females and spent a high proportion of their time above ground close to female burrows, partly far from their own burrows (C. Siutz, personal observation). Food caches in temporarily used burrows are very likely to be pilfered, and thus, males depend less strongly on food stores than females. In addition, previous findings have shown that body fat proportions at immergence are higher in adult males than adult females (Siutz et al. 2012). This supports the interpretation that adult males rely on internal rather than external energy reserves for hibernation. Females, in contrast, are more philopatric and use their burrows for longer periods, sometimes even the entire active and hibernation period (C. Siutz, personal observation). Accumulated food stores can, therefore, serve as energy reserves not only for the winter period but also during gestation and lactation. Female common hamsters can produce up to three litters per season, leading to extended reproductive periods in some individuals and limiting the time to prepare for the winter period (Franceschini-Zink and Millesi 2008; Siutz and Millesi 2012). The delayed hibernation onset in adult females, however, is not simply a conditional effect, because even females with relatively high immergence body mass also showed extended euthermic periods before entering the first torpor bout. Moreover, preliminary results on non-invasively calculated proportions of body fat and hibernation patterns indicate that females with relatively high body fat reserves also hibernated for shorter periods than males (Millesi et al. unpublished data).

In conclusion, the pronounced food hoarding behavior in females could enable them to cache larger amounts of food than males, resulting in sex-specific overwintering strategies in adult hamsters. If females adjusted the time spent hibernating in relation to their food stores, reduced torpor expression compared with males would be expected. Preliminary results of an experimental study on the use of torpor in relation to available food stores under laboratory conditions (Siutz et al. unpublished data) support this assumption: deep torpor bouts were more frequent in females that were prevented from building up food stores (but received small daily food rations) compared with others that had sufficient food reserves.

The quality and/or amount of remaining food stores after termination of hibernation could also affect the duration of preemergence euthermy. Hamsters with sufficient food stores could terminate hibernation earlier, allowing more time to prepare for the mating period. As common hamsters are reproductively active after their first hibernation, this is relevant for both yearling and older individuals (Lebl and Millesi 2008; Nechay et al. 1977; Vohralik 1974). Differences in stored food could explain why some individuals lost mass over winter, although the proportional losses were generally less than in hibernators without food stores (e.g., Arnold and Dittami 1997; Cochet et al. 1999; Fietz et al. 2005; Millesi et al. 1999b), whereas others left the hibernacula with a higher body mass than in the previous autumn. Mass loss did not differ in females and males. Thus, the higher energy costs caused by less time spent in the energy-saving torpor state must be compensated by sufficient food intake in the burrow. Male common hamsters usually had scrotal testes at vernal emergence, but testes size continued to increase until the onset of the mating period (Lebl and Millesi 2008). In our study, testes width at emergence strongly varied among individual males and was significantly related to the duration of preemergence euthermy: males with longer euthermic periods in the hibernaculum had larger testes. This underlines the importance of the preemergence euthermic period to activate the reproductive system without exposure to colder temperatures and predation risk outside the hibernaculum. In contrast to other species in which this period is more pronounced in males (Barnes 1996; Michener 1983, 1984, 1992; Munro et al. 2005; Young 1990), both male and female common hamsters showed similar durations of preemergence euthermy. The high individual variation in both groups is probably related to the availability of food reserves. The timing of reproduction can affect reproductive success in female common hamsters in that individuals which mated early in spring were able to produce more litters and more offspring compared with females with a longer latency to oestrus after vernal emergence (Franceschini-Zink and Millesi 2008). Almost all females emerged with an opened vagina, indicating that ovarian activity was initiated in the hibernaculum. Due to high dispersal rates, however, reproductive performance in the following active season could only be documented in six individuals. In these females, we found no relationship between preemergence euthermy and timing of conception in spring. Nevertheless, the absence of sex differences in the timing and duration of the euthermic phase prior to vernal emergence indicates that both sexes need this period after ending hibernation to attain reproductive maturation in spring.

Foraging strategies of juvenile common hamsters of both sexes resemble that of adult females, indicating the availability of external energy reserves (Tauscher and Millesi 2005). All juveniles studied here were born in spring or early summer; litters born late in the season were excluded. Thus, the temporal limits to build up food caches were less pronounced in our study animals compared with late-born juveniles. Juvenile hamsters immerged later than adult males, because juveniles need sufficient time to grow and prepare for hibernation (Michener 1984). We found no sex differences in the timing of immergence, hibernation onset, and body temperature patterns among juveniles, but juvenile males emerged earlier than the same-aged females. Considering that all individuals are reproductively active after their first hibernation, vernal emergence prior to females could enhance male mating opportunities. Our results may indicate that juveniles accumulated food stores in autumn, but consumed them mainly in spring during their preemergence period as reflected in the short duration of post-immergence compared with preemergence euthermy. In addition, the emergence body mass of yearlings was similar to older individuals of the same sex. Interestingly, adult individuals lost mass over winter, whereas juveniles on average showed a slight mass increase and could, therefore, catch up with older individuals. The combination of a relatively long hibernation duration and more food reserves than adult males seemed to allow juveniles, especially early born ones, to reach a similar body condition in spring as older conspecifics. This could enable some male yearlings to successfully compete for mates.

In conclusion, our results clearly show that the time spent inside the hibernaculum does not necessarily reflect the time spent in hibernation. All studied animals hibernated during the coldest period of the year from January until early March, when energy-saving strategies are most effective for survival. As in all hibernators, this is also reflected in the longest torpor bout durations at low ambient temperatures (e.g., Healy et al. 2012; Kart Gür and Gür 2015; Young 1990). The relatively long euthermic periods in the hibernacula are probably related to the opportunistic reproductive strategy of common hamsters, with early sexual maturity (in some individuals during their first season) and a mating period of up to 5 months (Berdyugin and Bolshakov 1998; Niethammer 1982; Seluga et al. 1996; Vohralik 1974). This leads to high reproductive effort, particularly in females. In field studies, the information on the quantity and quality of food stores is lacking. To shed light on the adjustment of hibernation in relation to external energy reserves in this species, experimental approaches with food supplements in the field and manipulations of food hoard availability and quality in the lab are required.
